# Hydrothermal Fabrication of Spindle-Shaped ZnO/Palygorskite Nanocomposites Using Nonionic Surfactant for Enhancement of Antibacterial Activity

**DOI:** 10.3390/nano9101453

**Published:** 2019-10-13

**Authors:** Aiping Hui, Shuqing Dong, Yuru Kang, Yanmin Zhou, Aiqin Wang

**Affiliations:** 1 Key Laboratory of Clay Mineral Applied Research of Gansu Province, Center of Eco-material and Green Chemistry, Lanzhou Institute of Chemical Physics, Chinese Academy of Sciences, Lanzhou 730000, China; aphui1215@163.com (A.H.); sqdong@licp.cas.cn (S.D.); yurukang@licp.cas.cn (Y.K.); 2 College of Animal Science and Technology, Nanjing Agricultural University, Nanjing 210095, China; zhouym6308@163.com (Y.Z.)

**Keywords:** ZnO, palygorskite, antibacterial agent, hydrothermal, nonionic surfactants

## Abstract

In order to improve the antibacterial performance of natural palygorskite, spindle-like ZnO/palygorskite (ZnO/PAL) nanocomposites with controllable growth of ZnO on the surface of PAL were prepared in the presence of non-ionic surfactants using an easy-to-operate hydrothermal method. The obtained ZnO/PAL nanocomposites have a novel and special spindle-shaped structure and good antibacterial activity against *Escherichia coli* (*E. coli*) and *Staphylococcus aureus* (*S. aureus*), and are also low cost. The minimum inhibitory concentrations of ZnO/PAL nanocomposites toward *E. coli* and *S. aureus* reached 1.5 and 5 mg/mL, respectively.

## 1. Introduction

With the rapid development of industry, antibiotics for animals have become more widely used in many countries. However, the widespread overuse of antibiotics with animals has been blamed for creating a potential threat for human beings [1,2,3], which poses a serious threat to the public health and stable global economic growth [4,5,6]. Global antimicrobial resistance was discussed at the G20 summit in Hangzhou, China. In this context, the use of antibiotics would be limited in consideration of the huge challenges of affordability and access of antimicrobials and their impact on public health. Therefore, it is urgent that a feasible and effective approach to prevent and reduce antibiotic resistance is explored [7].

Functional utilization of natural clay minerals and synthetic nanocomposites for antibacterial application has attracted great attention in academic and industrial research [8,9,10,11]. Clay minerals are a possible new answer to methicillin-resistant *Staphylococcus aureus* and other ‘superbug’ infections, and minerals found in clay deposits might be a good source of antibiotics that can combat superbugs [12,13]. Natural clay minerals that kill human pathogens could hold out hope for new economic uses and new drug designs, and the pH and oxidation state buffered by the clay mineral surfaces is key to controlling the solution chemistry and redox-related reactions occurring at the bacterial cell wall [12,14]. Therefore, it is expected that an effective way to solve antibiotic resistance by combining clay minerals and active antibacterial factors will be developed. Palygorskite (PAL), as a kind of clay minerals, is common in many parts of the world, typically forming in volcanic ash layers as rocks. However, the rods of natural PAL usually exist as bulk crystal bundles or aggregates because of van der Waals force, hydrogen bond, etc. In previous work, our groups have efficiently disaggregated the crystal bundles of PAL using high-pressure homogenization technology [15,16,17]. Although PAL displays low antibacterial activity, the highlight of PAL is focused on its carrier function of unique nanorods and nanochannels. For example, PAL is used as a carrier for loading of dodecyl triphenyl phosphonium bromide, which could be absorbed on the surface of PAL, the novel hybrid displays specific-targeting capability, long-term antibacterial activity and lower cytotoxicity [18]. PAL is also used as the carrier for adsorption of natural antibacterial agents such as ginger essential oil, where the content of ginger essential oil in the composite was estimated to be 18.66%. The composite had much higher antibacterial activity than single ginger essential oil and had specific-targeting antibacterial capability [19]. Meanwhile, natural PAL can support metal ions such as Ag, Cu and Zn ions to enhance the antibacterial and bioavailability activity [20,21]. Moreover, PAL loaded metal oxide such as zinc oxide (ZnO) serving as an inorganic antibacterial agent has been prepared using several methods including the coprecipitation technique, microwave-assisted decomposition, wet chemical method, sol-gel method and hydrothermal process [22,23,24,25,26].

Due to the growth of crystal diversity, controllability, nontoxicity and biosecurity, ZnO is considered to be an ideal material in designing new nanostructures [26,27,28,29,30,31,32]. The morphology-dependent antibacterial property of ZnO is an effective way to solve antibiotic resistance. The particular rod-like, needle-like and flower-like nanostructure displayed stronger physical damage and nanometer effect than common structures such as bulk and common ZnO [27,28,31,32,33]. These structures would lead to denaturation of membrane proteins, the permeability of membranes and further destruction of bacterial cell walls. PAL as a carrier to construct ZnO/PAL nanocomposites could prevent the aggregation of ZnO and enhance the synergistic effect of the obtained graded nanostructures, thus improving the antibacterial performance of PAL. However, the loading capacity of ZnO on the surface of PAL was very low, which limited the application of PAL as a antibacterial material [34]. Meanwhile, surfactant-assisted hydrothermal is very effective to control the growth of metallic oxide [35]. Herein, spindle-shaped ZnO/PAL nanocomposites were fabricated using a facile hydrothermal method in the presence of nonionic surfactant. The morphologies and compositions of the as-prepared samples were characterized, and a possible formation mechanism was proposed. Furthermore, the antibacterial activity of ZnO/PAL nanocomposites was determined using gram-negative *E. coli* and gram-positive *S. aureus* as model bacteria.

## 2. Materials and Methods

### 2.1. Materials

Natural PAL was provided by Huida Mineral Technology Co. Ltd., Jiangsu, China, and the main chemical compositions were 50.18% SiO_2_, 11.15% Al_2_O_3_, 4.21% MgO, 9.37% Fe_2_O_3_, 5.92% Na_2_O, 2.03% K_2_O and 1.37% CaO as determined using an X-ray fluorescence spectrometer (PANalytical Co., MiniPal 4, Almelo, The Netherlands). Zinc nitrate hexahydrate (Zn(NO_3_)_2_∙6H_2_O, 99.0%) was obtained from Tianjing Kermel Chemical Regent Company. Sodium hydroxide (NaOH, 96.0%) and Span 40 were purchased from China National Medicines Co., Ltd., Beijing, China. Tween 20 was bought from Xilong Chemical Co., Ltd., Shantou, China. Span 40 and Tween 20 were chemically pure, while other used reagents were analytical grade. All of chemical reagents were used without further purification, and distilled water was used throughout the experiment.

### 2.2. Preparation of ZnO/PAL Nanocomposites

Natural PAL was crushed and purified using 2% H_2_SO_4_ solution with a solid/liquid ratio of 1:10 deionized water solution corresponding to PAL mass to remove the associated carbonates, and the purified PAL was filtered by passing through a 200-mesh sieve for further use. ZnO/PAL nanocomposites were fabricated using a hydrothermal process. Typically, 15 mM of Zn(NO_3_)_2_∙6H_2_O, 2.7 g of PAL and 225 mM of NaOH were dissolved into 130 mL of deionized water. This was constantly stirred for 30 min, and 1 wt% Span 40 or 1 wt% Tween 20 corresponding to PAL was added into above solution, respectively. The mixture was ultrasonically dispersed for 60 min and then transferred into a 100 mL Teflon-lined stainless-steel autoclave, which was heated to 180 °C with a heating rate of 10 °C/min and maintained for 180 min. After being cooled to room temperature, the solid powder was collected using centrifugation and dried at 60 °C in an oven. The samples were labeled as ZnO/PAL-1 and ZnO/PAL-2, respectively. As a control, ZnO/PAL was prepared without adding the above nonionic surfactant. The optimized content of Span 40 and Tween 20 are shown in Supplementary Materials.

### 2.3. Characterization

X-ray diffraction (XRD) patterns of the obtained samples were acquired on X’pert PRO diffractometer (PAN analytical Co., Almelo, The Netherland) with Cu Kα radiation, 30 mA and 40 kV (*λ* = 1.54060 Å) at a scanning rate of 0.02° per second in the 2*θ* range from 3° to 80°. The morphologies of samples were observed on a field emission scanning electron microscopy (FE-SEM, JSM–6701F, JEOL, Tokyo, Japan) and a transmission electron microscope (TEM, JEM-1200EX, FEI, Hillsboro, OR, USA). Fourier transform infrared (FTIR) spectra of the samples were recorded on a Nicolet NEXUS spectrometer using potassium bromide pellets. The specific surface area (*S*_BET_) of the samples was evaluated via N_2_ adsorption, using Brunauer‒Emmett‒Teller analysis (BET, Micromeritics, Norcross, GA, USA). The values of *S*_BET_ were calculated using the BET equation, and the total pore volume (*V*_total_) was obtained from the volume of N_2_ held at the relative pressure *P*/*P*_0_ = 0.95. The surface area of the micropores (*S*_micro_) and the external surface area (*S*_ext_) were evaluated using the t-plot method. The ZnO loading content on PAL was determined using atomic absorption spectrometer ContrAA 700 (AAS, Analytik Jena AG, Jena, Germany). Zeta potential was measured on a Nano-ZS model zetasizer instrument (ZEN3600, Malvern, UK).

### 2.4. Antibacterial Assay

*Escherichia coli* (*E. coli*) and *Staphylococcus aureus* (*S. aureus*), kindly provided by China Veterinary Culture Collection Center, were tested as representative culture both gram-negative and gram-positive bacteria. The antibacterial activity of various ZnO/PAL nanocomposites was evaluated by examining the minimum inhibitory concentration (MIC), as shown in Figure S1 (Supplementary Materials). *E. coli* and *S. aureus* were isolated from the Luria‒Bertani (LB) agar plate, added into fresh LB broth separately for incubation at 37 °C in a shaking incubator at a speed of 160 r/min for 12 h. For future use, 1:100 of bacteria liquid and fresh medium (volume ratio) was inoculated to the fresh medium again and cultivated for 3 h into the logarithmic phase. A certain amount of sample mixed with 40 mL MacConkey medium using high pressure sterilization. When the MacConkey medium cooled to 50–60 °C, it was added to a sterilization culture dish to obtain various concentrations of 5, 2.5, 1.5 and 1 mg/mL (*E. coli*) and 10, 5, 2.5 and 1.5 mg/mL (*S. aureus*) medium, respectively. 1 μL of fresh bacteria liquid (10^8^ CFU/mL) was put into medium with three parallel dots at different locations, and three parallel plates were conducted for each sample. The plates were kept in an incubator at 37 °C for 24 h and the bacterial colonies were observed to test antibacterial performance. The MIC value was defined as the lowest concentration of ZnO/PAL nanocomposites when there was no visible bacterial colony. For the control, excluding antibacterial materials on agar plate as positive control in the same way, no samples and bacteria liquid dots were used as blank control. All experiments were conducted in duplicate, and also the MIC value was reported as the lowest concentration to inhibit completely the growth of each bacterial strain being tested.

## 3. Results and Discussion

### 3.1. FTIR

**Figure 1:**
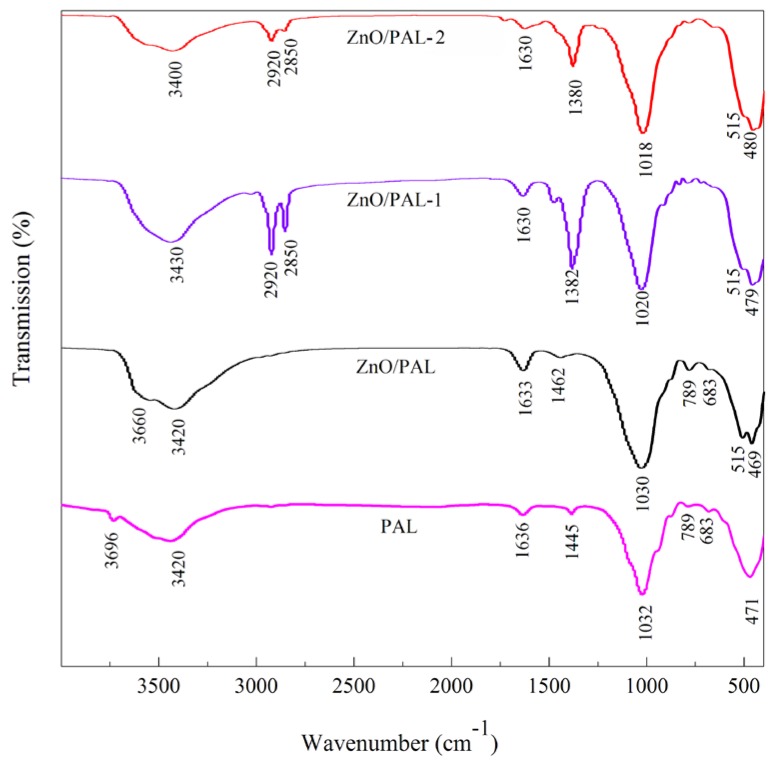
FTIR spectra of PAL, ZnO/PAL, ZnO/PAL-1 and ZnO/PAL-2.

To determine whether the Span 40 and Tween 20 were capable of inducing surface modification on ZnO/PAL or not, FTIR spectra were conducted. The FTIR spectra of PAL, ZnO/PAL, ZnO/PAL-1 and ZnO/PAL-2 are shown in Figure 1. The absorption bands at 3696 and 3420 cm^–1^ were ascribed to the –OH stretching vibration of Mg–OH groups, the stretching vibration of coordinated water and the stretching vibration of adsorbed water, respectively [36,37]. After the hydrothermal process and the loading of ZnO crystal, there is no clear change in the characteristic bands of Si–O stretching vibrations of PAL at 1194 and 1086 cm^–1^ [36]. By contrast, the new absorption band at 515 cm^–1^ was attributed to the stretching vibration of the Zn–O band of ZnO/PAL. After incorporation of Span 40 and Tween 20, the bands at 2850 and 2920 cm^–1^ were observed, which were attributed to the characteristic stretching vibration bands of CH_2_ derived from Span 40 and Tween 20 [38]. Meanwhile, it could be inferred that ZnO and a small amount of Span 40 or Tween 20 simultaneously was loaded and attached on the surface of PAL during the preparation process.

### 3.2. XRD Patterns

**Figure 2:**
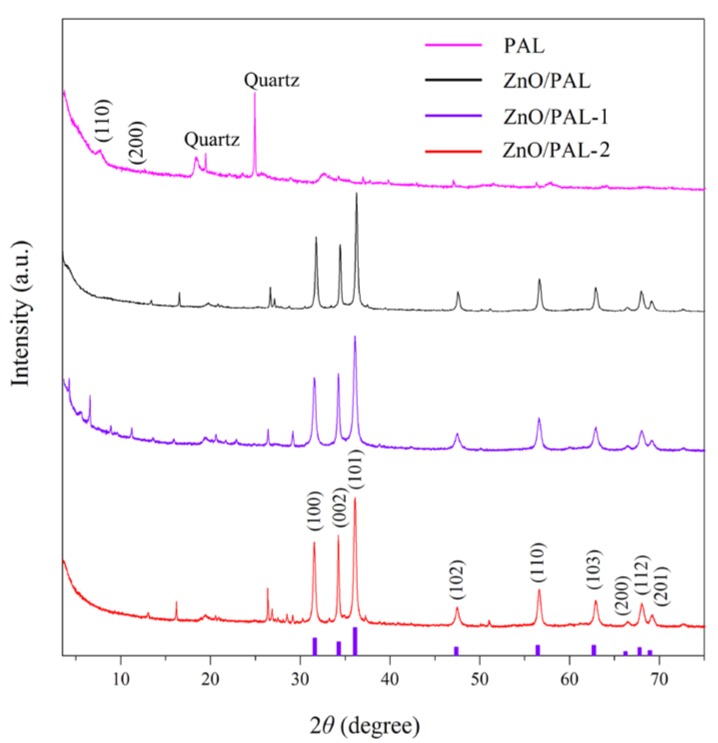
X-ray diffraction (XRD) patterns of PAL, ZnO/PAL, ZnO/PAL-1 and ZnO/PAL-2.

The crystalline structures of PAL and ZnO/PAL nanocomposites were characterized using XRD (Figure 2). In the XRD pattern of PAL, the diffraction peaks at 2*θ* of 8.38°, 13.74°, 16.34° and 34.38° were attributed to the characteristic peaks of PAL [37]. The diffraction peaks located at 2*θ* = 20.8° and 26.7° are the typical diffraction peaks of quartz [37]. After incorporation of ZnO, the diffraction peaks of ZnO were observed from the XRD patterns of the obtained ZnO/PAL at 2*θ* = 31.76°, 34.46°, 36.26°, 47.58°, 56.60°, 62.92°, 66.46°, 67.96° and 69.09°, which corresponded to the planes of (100), (002), (101), (102), (110), (103), (200), (112) and (201), respectively, indicating a wurtzite structure (JCPDS standard card 36-1451) [31]. In particular, it could be clearly seen that the (110) diffraction peak of PAL at 2*θ* = 8.38° weakened after hydrothermal reaction. The XRD pattern of ZnO/PAL-1 was not similar to that of ZnO/PAL and ZnO/PAL-1 nanocomposites, there was a characteristic peak at 2*θ* = 6.05° of ZnO/PAL-1 after the hydrothermal process, which might be ascribed to the (001) direction peak of smectite. This phenomenon might be attributed to the transformation of PAL into smectite at alkaline medium during the hydrothermal process [39]. The particle grain size of ZnO/PAL nanocomposites could be calculated based on the X-ray line broadening method using Scherrer’s equation:

(1) $$D=\frac{k\lambda }{\beta \cos\theta }$$

where *D* is the size of the nanometers, *λ* is the wavelength of the radiation (1.5406 Å for Cu Kα), k is a constant (0.94), *β* is the peak width at half-maximum intensity and *θ* is the peak position. The average crystallite sizes of ZnO/PAL, ZnO/PAL-1 and ZnO/PAL-2 were 63.1, 54.6 and 52.5 nm, respectively. The difference in the crystallite size might be mainly derived from the incorporation of surfactant during the hydrothermal process, which might affect the nucleation and the subsequent growth rate of ZnO crystal.

### 3.3. SEM and TEM

**Figure 3:**
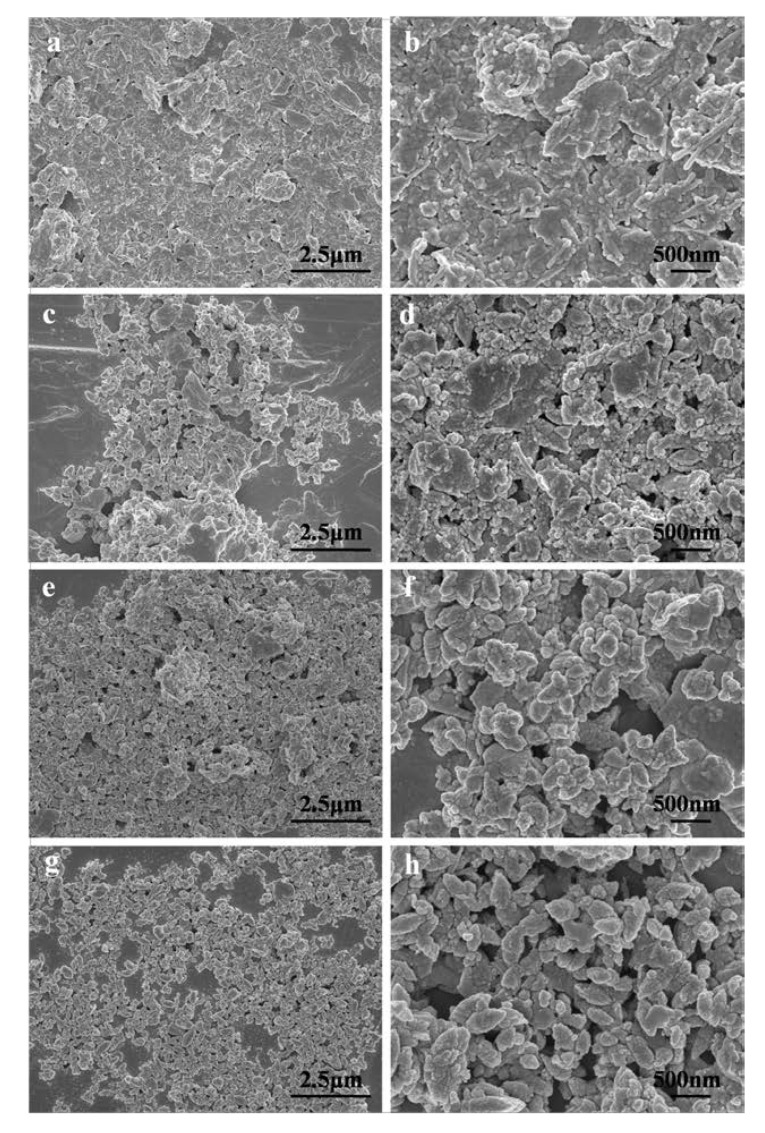
Field emission scanning electron microscopy (FE-SEM) images of (**a, b**) PAL, (**c, d**) ZnO/PAL, (**e, f**) ZnO/PAL-1 and (**g, h**) ZnO/PAL-2.

The morphologies of PAL, ZnO/PAL, ZnO/PAL-1 and ZnO/PAL-2 are provided in Figure 3. As depicted in Figure 3a,b, the rod-like structure of PAL was clearly observed and the enlarged SEM image can be seen in Supplementary Materials (Figure S2). The rod of PAL disappeared after the hydrothermal process, and an irregular morphology appeared (Figure 3c,d), which was consistent with the XRD results of the crystal phase transition of PAL. With the addition of nonionic surfactants, the morphologies of the obtained samples changed clearly. It could be seen that different nonionic surfactants played a significant role in the morphologies of the ZnO/PAL nanocomposites. In the case of Span 40 (Figure 3e,f), irregular morphology was obtained, while the a spindle-shape morphology was clearly observed after addition of Tween 20 (Figure 3g,h). In addition, the composite showed good dispersion, and no clear agglomeration was observed after adding nonionic surfactant.

**Figure 4:**
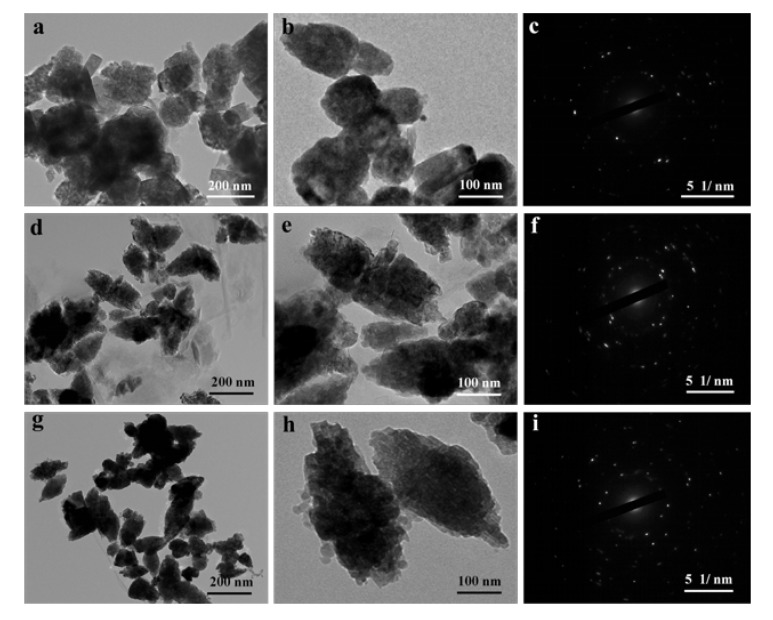
Transmission electron microscope (TEM) images and selected area electron diffraction pattern of (**a**–**c**) ZnO/PAL, (**d**–**f**) ZnO/PAL-1 and (**g**–**i**) ZnO/PAL-2.

In addition, the as-prepared samples were also studied using TEM. As shown in Figure S3 (Supplementary Materials), the PAL had a typical rod-like structure with a length of 250−500 nm and a diameter of about 40 nm. After the hydrothermal process and loading of ZnO, the ZnO/PAL nanocomposites indicated a disordered structure (Figure 4a,b), which was mainly ascribed to the crystal phase transition of PAL at alkaline medium during the hydrothermal process [39]. By contrast, the introduction of nonionic surfactants was in favor of adjusting the assembly of ZnO nanoparticles, especially Tween 20. TEM images of ZnO/PAL-Tween 20 nanocomposites displayed a typical spindle-shaped structure (Figure 4g,h), which was consistent with the result of SEM. There was not an obvious spindle-shaped structure (Figure 4d,e), but the observable structures were all spindle-like structures in Figure 4g,h, and the structures were more uniform and complete. Furthermore, the corresponding selected area electron diffraction pattern, exhibited in Figure 4c,f,i, indicated the samples had a single polycrystalline structure.

Furthermore, the effect of the loading of ZnO on PAL also could be confirmed by the changes of the pore structural parameters. As shown in Table 1, the *S*_BET_ of PAL was 173.5 m^2^/g, while the *S*_BET_ of ZnO/PAL decreased to 35.4 m^2^/g. This phenomenon was mainly related to the loading of ZnO and the crystal phase transition of PAL. It should be noted that the *S*_BET_ values of ZnO/PAL-1 and ZnO/PAL-2 decreased drastically to 8.9 and 14.2 m^2^/g, respectively, which might be attributed to the anchoring of ZnO on PAL with the addition of Span 40 and Tween 20.

### 3.4. Possible Formation Mechanism of ZnO/PAL Nanocomposites

**Figure 5:**
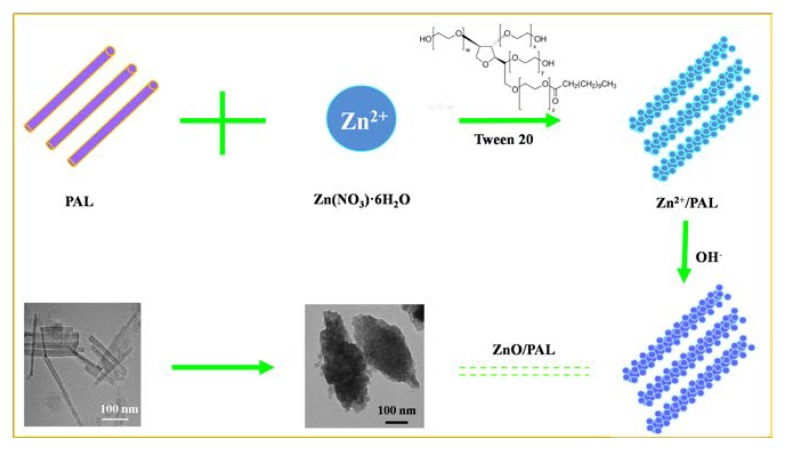
Schematic illustration for the preparation of spindle-like ZnO/PAL.

The preparation procedure of ZnO/PAL nanocomposites is illustrated in Figure 5. It is well-known that PAL has a permanent negative charge (Figure S4, Supplementary Materials); therefore, due to the charge between the PAL and Zn^2+^, the positive Zn^2+^ could be adsorbed on the surface of rod-like PAL. Then, the adsorbed Zn^2+^ was transformed into Zn(OH)_2_ on the surface of PAL with the increasing of OH^−^. Thereafter, Zn(OH)_2_ was changed into ZnO crystals after the hydrothermal process accompanied with the crystal phase transition of PAL. Moreover, the addition of Tween 20 was a necessary step for the formation of spindle-shaped ZnO/PAL nanostructures. During the hydrothermal process, the nucleation and seed growth of ZnO occurred, and subsequently, the generated ZnO assembled on the surface of rod-like PAL. In the following step, the excess Tween 20 played an important role in the assembly of ZnO crystals on the PAL surface. When Tween 20 was dissolved into aqueous solution, it reduced both the dielectric constant of water and the surface tension of ZnO crystals. The “surface-solvent effect” influenced the growth and deposition of ZnO seeds on PAL. The deposition and growth rate of ZnO seeds in the middle were faster than at the two ends of PAL; thus, spindle-like structures appeared [27,31]. Therefore, it was suggested that the suitable surfactant-assisted process might be an easy way for the preparation of a particular structure of ZnO/PAL nanocomposites.

### 3.5. Antibacterial Evaluation

**Figure 6:**
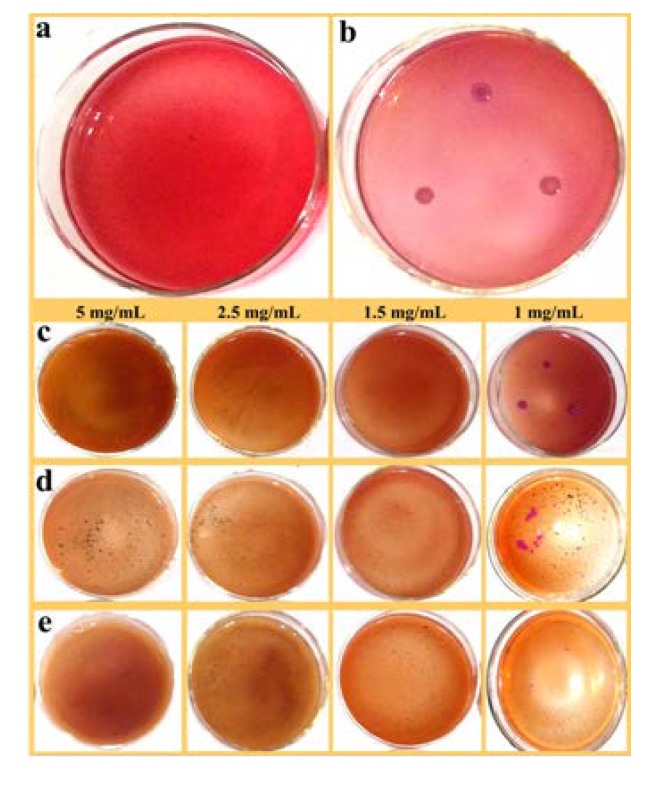
(**a**) Blank control, (**b**) positive control of *E. coli* and *E. coli* treated using (**c**) ZnO/PAL, (**d**) ZnO/PAL-1 and (**e**) ZnO/PAL-2.

The antibacterial activity of ZnO/PAL, ZnO/PAL-1 and ZnO/PAL-2 was determined using gram-negative bacterium *E. coli* and gram-positive bacterium *S. aureus* as model bacteria. Figure 6a,b were blank control and positive control of *E. coli*, respectively. It was observed that the bacteria could be detected among a series of plates with the contact of ZnO/PAL (Figure 6c). However, there was no obvious change when the model bacteria were treated using PAL; the MIC value of PAL was more than 50 mg/mL (Figure S5, see Supplementary Materials). The results further indicated that deposited ZnO on rod-like PAL was a feasible method to realize the functional utilization of natural PAL mineral as an antibacterial agent. Interestingly, a few plate appeared *E. coli* colonies after being contacted with ZnO/PAL-1 (Figure 6d), and when the concentration of ZnO/PAL-2 was 1mg/mL, there was *E. coli* colonies in the plate (Figure 6e). The MIC values of ZnO/PAL, ZnO/PAL-1 and ZnO/PAL-2 against *E. coli* were 5 mg/mL, 2.5 mg/mL and 1.5 mg/mL, respectively (Table 2).

**Figure 7:**
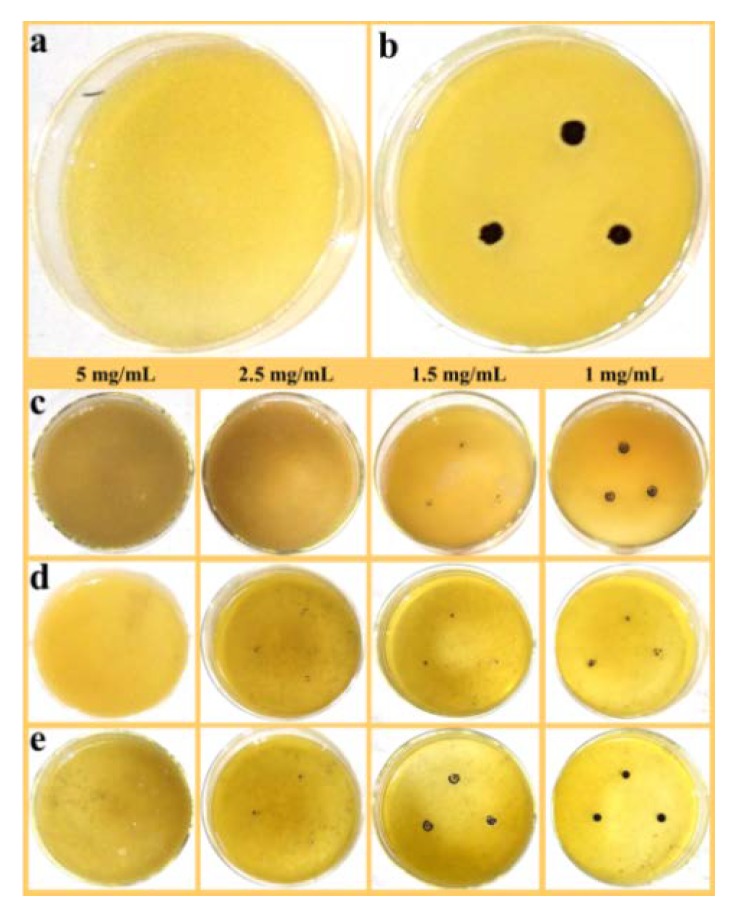
(**a**) Blank control, (**b**) positive control of *S. aureus* and *S. aureus* treated using (**c**) ZnO/PAL, (**d**) ZnO/PAL-1 and (**e**) ZnO/PAL-2.

Figure 7a,b show the blank control and positive control of *S. aureus*, respectively. Similarly, the MIC value of PAL toward *S. aureus* was greater than 50 mg/mL (Figure S6, see Supplementary Materials), the results for *S. aureus* displayed the same trend of inhibiting colonies as the concentration decreased. In brief, the MIC values of ZnO/PAL, ZnO/PAL-1 and ZnO/PAL-2 against *S. aureus* were 2.5, 5 and 5 mg/mL (Table 2). As a comparative study, the ZnO/PAL was prepared with various contents of Span 40 and Tween 20, and the MIC values are presented in Tables S1 and S2 (Supplementary Materials).

Compared with the PAL and ZnO/PAL samples, ZnO/PAL-1 and ZnO/PAL-2 showed much better antibacterial activity towards *E. coli*. These results indicated that surfactant-assisted synthesis of spindle-like ZnO/PAL displayed an obvious effect on antibacterial performance against *E. coli*, especially Tween 20 assisted synthesis of ZnO/PAL. It is worth mentioning that the formation of spindle-shaped nanostructure might be the prime reason for the enhanced antibacterial activity of ZnO/PAL-2 against *E. coli*. Moreover, the surface charge of the spindle-like ZnO/PAL was another possible factor in affecting the antibacterial property. In order to further verify this hypothesis, the zeta potential of the samples was conducted (Figure S5, see Supplementary Materials). The zeta potentials of PAL, ZnO/PAL, ZnO/PAL-1 and ZnO/PAL-2 were −16.2, −20.2, −26.8 and −21.0 mV, respectively. It is worth noting that the surface charge of the model bacterium is electronegativity, and the sulfur and oxygen groups contained unshared electron pairs, which played a role in the bactericidal process [33,40]. All of the samples exhibited negative charge with the different values, which might exhibit some differences in the killing efficiency toward different kinds of bacteria. 

More interestingly, the obtained samples showed different antibacterial activity against *E. coli* and *S. aureus* under the same condition, and also the same samples (ZnO/PAL-2) exhibited better antibacterial activity towards *E. coli* than *S. aureus*. The difference of antibacterial effect between two kinds of microorganisms may well be attributed to the different structures and chemical compositions of the cell surfaces [9,10,40]. The cell wall composition of *E. coli* is protein and lipopolysaccharide, with a loose structure and a thickness of generally 10 to 13 nm. Therefore, *E. coli* is not easily able to resist external damage. In contrast, the main ingredients of gram-positive bacteria *S. aureus* are teichoic acid and peptidoglycan. The cell wall is thick, in the range of 20−80 nm; thus, the *S. aureus* presents better defense properties against the external mechanical injury and maintained a complete cell shape [3].

In addition, the antibacterial process of ZnO/PAL nanocomposites was a complex reaction, comprising the generation of reactive oxygen species such as ∙OH, ∙O_2_^−^, ^1^O_2_ and H_2_O_2_ [7,31,41]. The generation of reactive oxygen species was attributed to the production of photoinduced charge carriers and the interaction with oxygen and water molecules on the surface of ZnO/PAL nanocomposites [27,39]. The antibacterial property of ZnO/PAL nanocomposites was mainly due to the production of hydroxyl radicals [3,42,43]. Herein, PAL served as carrier to load ZnO, and the obtained samples exhibited a smaller size and rod-like or spindle-shaped nanostructure, which might be the main reason for the improved antibacterial performance. The spindle-shaped nanostructure could damage the wall of the cell, which inhibited the growth of microorganisms. The smaller end of the spindle-shaped ZnO/PAL exhibited stronger mechanical damage and nano effect than PAL. When the concentration of PAL and ZnO/PAL suspensions was similar, especially with little difference in ZnO loading content (Table S3, see Supplementary Materials), it is easier for the smaller sized, spindle-shaped ZnO/PAL nanocomposites to diffuse and adhere to the surface of a microorganism cell wall or membrane. The particular structure might lead to the denaturation of membrane proteins and then change the permeability of the membrane and further destroy the microorganism cell wall or membrane structure [27,28,42]. The shape-dependent antibacterial activity of ZnO/PAL-2 was explained in terms of the percent of active facets; high-atom-density facets exhibit higher antibacterial activity. The spindle-shaped structures of ZnO/PAL have more active facets; the size of the end part of the spindle-like structure is much smaller than the middle so that increases the exposure and activity of the ZnO crystal, which leads to better antibacterial property [43,44].

## 4. Conclusions

In summary, ZnO/PAL nanocomposites were successfully prepared using a simple hydrothermal method in the presence of non-ionic surfactants. The addition of nonionic surfactants, especially Tween 20, played an important role in regulating the morphologies of ZnO/PAL nanocomposites. The MIC values of the spindle-shaped ZnO/PAL nanocomposites against *E. coli* and *S. aureus* were 1.5 and 5 mg/mL, respectively. On this basis, it is expected that a new type of antibacterial material will be developed by combining natural clay minerals with inorganic antibacterial agents, which could effectively reduce the production cost of inorganic antibacterial agents and realize the high value application of natural clay minerals.

## Figures and Tables

**Table 1:** *S*_BET_, *S*_micro_, *S*_ext_ and *V*_total_ of PAL, ZnO/PAL, ZnO/PAL-1 and ZnO/PAL-2.

Samples	*S*_BET_ (m^2^/g)	*S*_micro_ (m^2^/g)	*S*_ext_ (m^2^/g)	*V*_total_ (cm^3^/g)
PAL	173.5	39.3	134.2	0.197
ZnO/PAL	35.4	2.4	32.3	0.067
ZnO/PAL-1	8.9	−	10.7	0.025
ZnO/PAL-2	14.2	−	22.0	0.036

**Table 2:** The minimum inhibitory concentration (MIC) values of PAL, ZnO/PAL, ZnO/PAL-1 and ZnO/PAL-2 against *E. coli* and *S. aureus*.

Samples	MIC (mg/mL)							
	*E. coli*				*S. aureus*			
	5	2.5	1.5	1	5	2.5	1.5	1
PAL	✕	✕	✕	✕	✕	✕	✕	✕
ZnO/PAL	✓	✕	✕	✕	✓	✓	✕	✕
ZnO/PAL-1	✓	✓	✕	✕	✓	✕	✕	✕
ZnO/PAL-2	✓	✓	✓	✕	✓	✕	✕	✕

✓―The sample could inhibit completely the growth of each bacterial strain. ✕ ―The sample could not inhibit completely the growth of each bacterial strain.

## Supplementary Materials

The following are available online at https://www.mdpi.com/2079-4991/9/10/1453/s1, Figure S1: Schematic illustration for the antibacterial test, Figure S2: SEM image of PAL, Figure S3: TEM image of PAL, Figure S4: Zeta potential of PAL, ZnO/PAL, ZnO/PAL-1 and ZnO/PAL-2, the pH was 6.3 (aqueous solution), Figure S5: (a) Blank control, (b) positive control of *E. coli*, *E. coli* treated by PAL with various concentrations of (c) 50 mg/mL, (d) 20 mg/mL, (e) 10 mg/mL, (f) 1 mg/mL, Figure S6: (a) Blank control, (b) positive control of *S. aureus*, *S. aureus* treated by PAL with various concentrations of (c) 50 mg/mL, (d) 20 mg/mL, (e) 10 mg/mL, (f) 1 mg/mL, Table S1: The MIC values of ZnO/PAL nanocomposites prepared by different content of Span 40 against *E. coli* and *S. aureus*, Table S2: The MIC values of ZnO/PAL nanocomposites prepared by different content of Tween 20 against *E. coli* and *S. aureus*, Table S3: The ZnO loading of ZnO/PAL-1 and ZnO/PAL-2 determined by AAS.
