# Supplementary material for: Hydrothermal Fabrication of Spindle-Shaped ZnO/Palygorskite Nanocomposites Using Nonionic Surfactant for Enhancement of Antibacterial Activity
Source: Nanomaterials (Basel). 2019 Oct 13;9(10):1453. doi: 10.3390/nano9101453 (PMC6836143; doi:10.3390/nano9101453)
Supplement: Supplementary file 1 [file nanomaterials-09-01453-s001.pdf]

nanomaterials

MDPI

Supplementary Materials

Hydrothermal Fabrication of Spindle-Shaped ZnO/Palygorskite Nanocomposites Using Nonionic Surfactant for Enhancement of Antibacterial Activity

Aiping Hui [1], Shuqing Dong [1], Yuru Kang [1], Yanmin Zhou [2] and Aiqin Wang [1,*]

1 Key Laboratory of Clay Mineral Applied Research of Gansu Province, Center of Eco-material and Green Chemistry, Lanzhou Institute of Chemical Physics, Chinese Academy of Sciences, Lanzhou 730000, China; aphui1215@163.com (A.H.); sqdong@licp.cas.cn (S.D.); yurukang@licp.cas.cn (Y.K.)

2 College of Animal Science and Technology, Nanjing Agricultural University, Nanjing 210095, China; zhouym6308@163.com (Y.Z.)

* Correspondence: aqwang@licp.cas.cn; Tel.: +86-931-4868-118

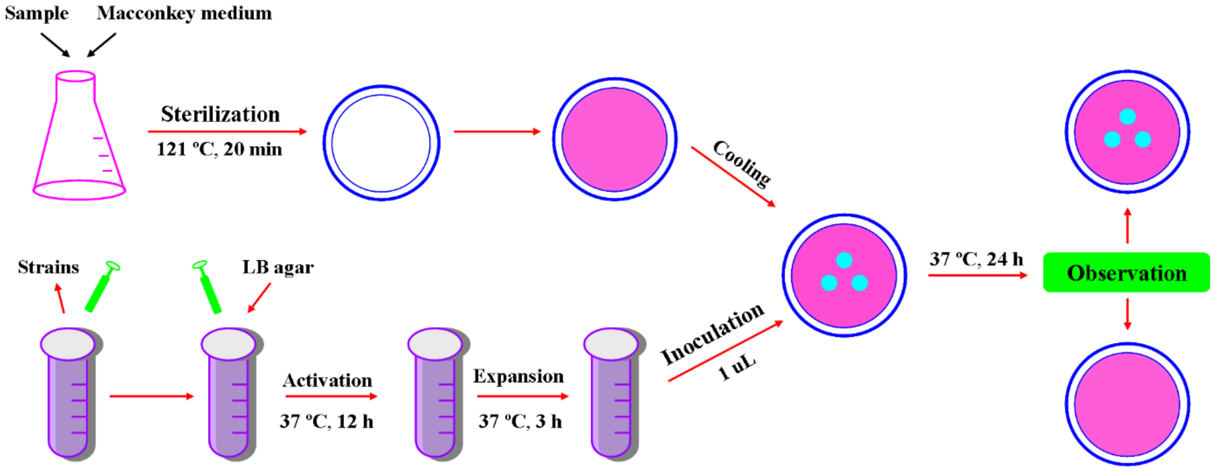

Figure S1. Schematic illustration for the antibacterial test.

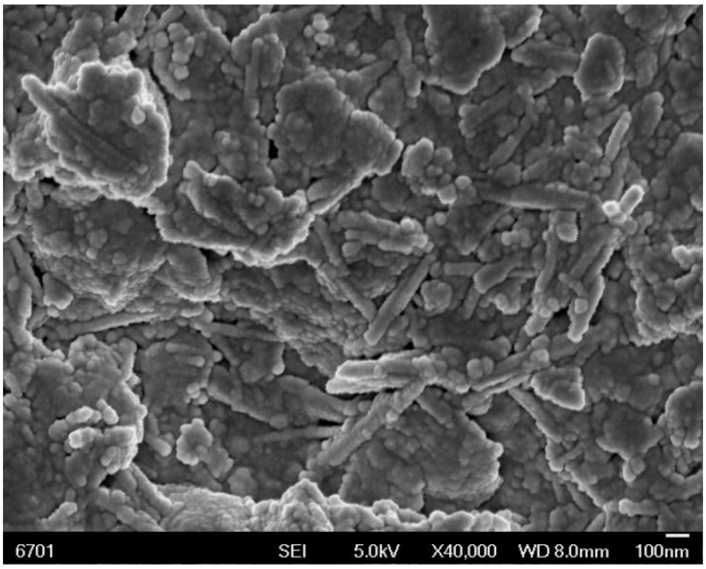

Figure S2. SEM image of PAL.

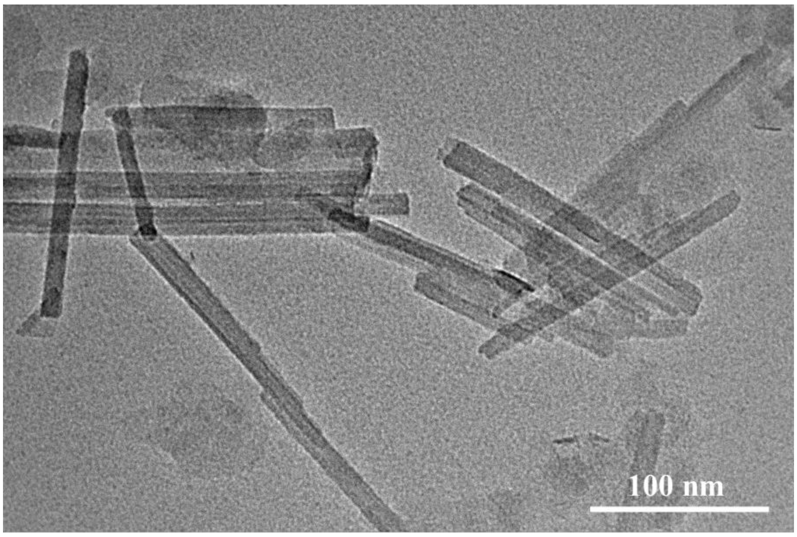

Figure S3. TEM image of PAL.

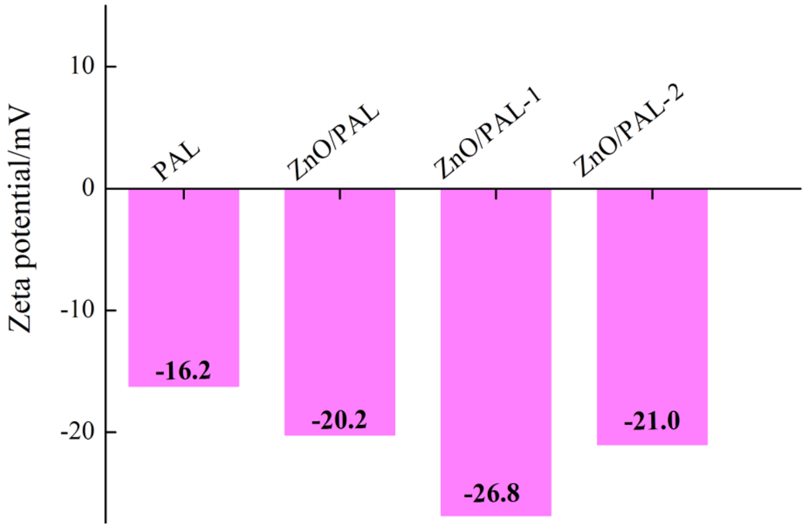

Figure S4. Zeta potential of PAL, ZnO/PAL, ZnO/PAL-1 and ZnO/PAL-2, the pH was 6.3 (aqueous solution).

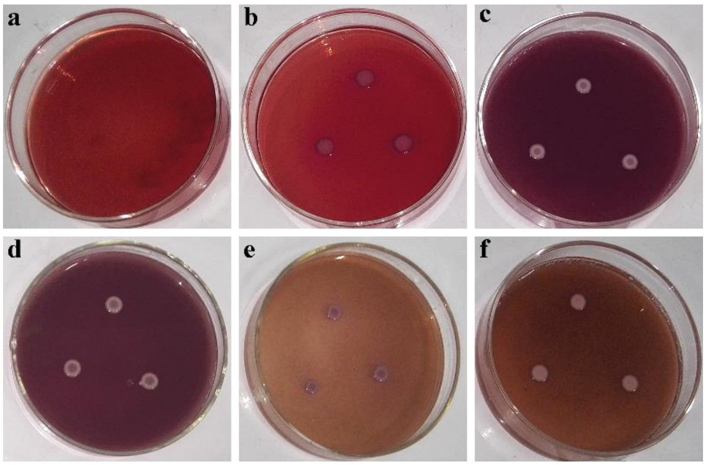

Figure S5. (a) Blank control, (b) positive control of *E. coli*, *E. coli* treated by PAL with various concentrations of (c) 50 mg/mL, (d) 20 mg/mL, (e) 10 mg/mL, (f) 1 mg/mL.

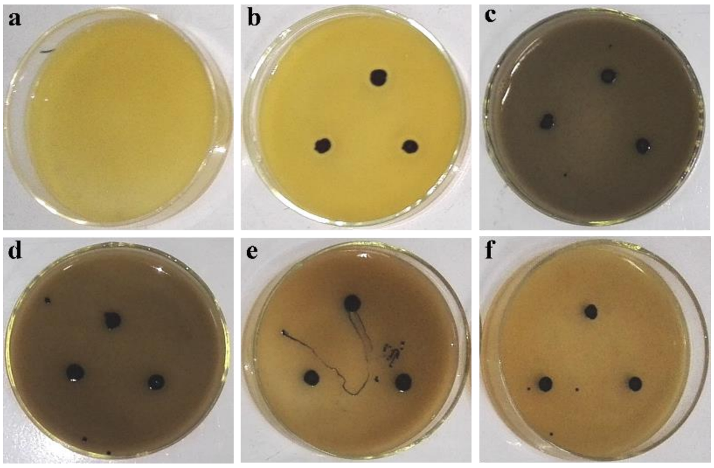

Figure S6. (**a**) Blank control, (**b**) positive control of *S. aureus*, *S. aureus* treated by PAL with various concentrations of (**c**) 50 mg/mL, (**d**) 20 mg/mL, (**e**) 10 mg/mL, (**f**) 1mg/mL.

Preparation of ZnO/PAL Nanocomposites

Natural PAL was crushed and purified by 2% H_2SO_4 solution with a solid/liquid ratio of 1:10 deionized water solution corresponding to PAL mass to remove the associated carbonates, and the purified PAL was filtered by passing through a 200-mesh sieve for further use. ZnO/PAL nanocomposites were fabricated by hydrothermal process. Typically, 15 mM $Zn(NO_3)_2{\cdot}6H_2O$ and 2.7 g PAL and 225 mM NaOH were dissolved into 130 mL deionized water. Constant stirring for 30 min, different content (0.25, 0.5, 1.5, 2.0 wt%) of Span 40 and (0.25, 0.5, 1.5, 2.0 wt%) of Tween 20 corresponding to PAL were added into above solution, respectively. The mixture was ultrasonically dispersed for 60 min and then transferred into a 100 mL Teflon-lined stainless-steel autoclave, which was heated to 180 °C and maintained for 180 min. After being cooled to room temperature, the solid powder was collected by centrifugation and dried at 60 °C in an oven.

Table S1. The MIC values of ZnO/PAL nanocomposites prepared by different content of Span 40 against *E. coli* and *S. aureus*.

Content of Span 40 (%)	MIC (mg/mL)							
	E. coli				*S. aureus*			
	5	2.5	1.5	1	5	2.5	1.5	1
0.25	✓	×	×	×	×	×	×	×
0.5	✓	✓	×	×	✓	×	×	×
1.5	✓	×	×	×	✓	×	×	×
2	✓	×	×	×	✓	×	×	×

✓—The sample could inhibit completely the growth of each bacterial strain

×—The sample could not inhibit completely the growth of each bacterial strain

Table S2. The MIC values of ZnO/PAL nanocomposites prepared by different content of Tween 20 against *E. coli* and *S. aureus*.

Content of Tween 20 (%)	MIC (mg/mL) *E. coli*				*S. aureus*			
	5	2.5	1.5	1	5	2.5	1.5	1
0.25	×	×	×	×	×	×	×	×
0.5	✓	✓	×	×	✓	×	×	×
1.5	✓	✓	×	×	✓	×	×	×
2	✓	✓	×	×	✓	×	×	×

✓—The sample could inhibit completely the growth of each bacterial strain.
×—The sample could not inhibit completely the growth of each bacterial strain.

Table S3. The ZnO loading of ZnO/PAL-1 and ZnO/PAL-2 determined by AAS.

Samples	Zn Content (%)	ZnO loading content (%)
ZnO/PAL-1	32.98	41.09
ZnO/PAL-2	32.75	40.81
